# Dimerization Interface of 3-Hydroxyacyl-CoA Dehydrogenase Tunes the Formation of Its Catalytic Intermediate

**DOI:** 10.1371/journal.pone.0095965

**Published:** 2014-04-24

**Authors:** Yingzhi Xu, He Li, Ying-Hua Jin, Jun Fan, Fei Sun

**Affiliations:** 1 National Laboratory of Biomacromolecules, Institute of Biophysics, Chinese Academy of Sciences, Beijing, China; 2 Key Laboratory for Molecular Enzymology & Engineering of the Ministry of Education, Jilin University, Changchun, China; 3 University of Chinese Academy of Sciences, Beijing, China; 4 Department of Physics and Materials Science, City University of Hong Kong, Hong Kong SAR, China; Russian Academy of Sciences, Institute for Biological Instrumentation, Russian Federation

## Abstract

3-hydroxyacyl-CoA dehydrogenase (HAD, EC 1.1.1.35) is a homodimeric enzyme localized in the mitochondrial matrix, which catalyzes the third step in fatty acid *β*-oxidation. The crystal structures of human HAD and subsequent complexes with cofactor/substrate enabled better understanding of HAD catalytic mechanism. However, numerous human diseases were found related to mutations at HAD dimerization interface that is away from the catalytic pocket. The role of HAD dimerization in its catalytic activity needs to be elucidated. Here, we solved the crystal structure of *Caenorhabditis elegans* HAD (*c*HAD) that is highly conserved to human HAD. Even though the *c*HAD mutants (R204A, Y209A and R204A/Y209A) with attenuated interactions on the dimerization interface still maintain a dimerization form, their enzymatic activities significantly decrease compared to that of the wild type. Such reduced activities are in consistency with the reduced ratios of the catalytic intermediate formation. Further molecular dynamics simulations results reveal that the alteration of the dimerization interface will increase the fluctuation of a distal region (a.a. 60–80) that plays an important role in the substrate binding. The increased fluctuation decreases the stability of the catalytic intermediate formation, and therefore the enzymatic activity is attenuated. Our study reveals the molecular mechanism about the essential role of the HAD dimerization interface in its catalytic activity via allosteric effects.

## Introduction

3-Hydroxyacyl-CoA dehydrogenase (EC 1.1.1.35) is the penultimate enzyme in fatty acid *β*-oxidation (FAO) cycle, catalyzing the oxidation of L-3-hydroxyacyl-CoA to 3-ketoacyl-CoA concomitant with the reduction of NAD^+^ to NADH (nicotinamide adenine dinucleotide reduced form) [1]. The 3-hydroxyacyl-CoA dehydrogenase enzyme family consists of three members according to their specificity on the substrate chain length: the long-chain (C10∼C16), short-chain (shorter than C4) and medium/short chain (C4∼C10) 3-hydroxyacyl-CoA dehydrogenases. Unlike the long-chain 3-hydroxyacyl-CoA dehydrogenase (LCHAD) that is a component of membrane-associated multifunction protein in mitochondrial and peroxisome [2], [3], the short-chain 3-hydroxyacyl-CoA dehydrogenase (SCHAD) and medium/short chain 3-hydroxyacyl-CoA dehydrogenase (M/SCHAD) reside in the mitochondrial matrix [4], [5]. However, M/SCHAD (hereafter named as HAD, see [6]), rather than SCHAD, provides the majority of 3-hydroxyacyl-CoA dehydrogenase activity in mitochondria, due to its higher expression level [5], [7] and broader chain length catalytic activity [8].

Human HAD deficiency is a recessively inherited disorder of fatty acid metabolism with various clinical presentations including hypertrophic cardiomyopathy, hypoketotic hypoglycemia, skeletal myopathy, and liver dysfunction [9]. Vredendaal *et al*. mapped human *HAD* gene to chromosome 4q22-26 and further described the structure organization of the gene to encompass 8 exons and 7 introns [10], [11]. Disease-causing mutations of *HAD* gene were continuously reported in the past decades, which encode mutant HAD proteins with a significantly decreased activity [12]–[18]. Their clinical manifestations and mutation sites are summarized in **Table 1**.

**Table 1:** Reported disease-causing mutations of human *HAD* gene.

Mutation Site	Allele	Year reported	Clinical manifestation	Reference
P246L^a^	Homozygous	2001	HH^e^	[13]
c.547-3_549del^b^	Homozygous	2004	HH^e^	[14]
IVS6-2a>g^c^	Homozygous	2005	HH^e^ and seizures	[15]
D45G/Y214H	Compound heterozygous	2006	Reye syndrome but not HI^f^	[16]
M176V^a^	Homozygous	2009	Protein induced HH^e^	[17]
R224X^a^^,^ d	Homozygous	2009	HH^e^	[18]

a Number of the mutant residue was revised with the exclusion of transit peptide as compared with original literature.

b This deletion mutation affected RNA splicing and lead to a mRNA lacking exon 5.

c This mutation locates in splice site and the resulting mRNA were shown to comprise abnormal exon 7 sequence.

d The codon encoding Arg (CGA) was mutated to STOP codon (TGA) at position 224.

e Hyperinsulinemic hypoglycemia.

f Hyperinsulinism.

The crystal structure of human HAD [19], [20] consists of two domains, the N-terminal domain and the C-terminal domain. The former one (catalytic domain) resembles an α/β dinucleotide-binding fold (Rossmann-fold) and comprises a conserved His-Glu pair in the active site. The latter one (dimerization domain) is primarily α-helical and contributes to the HAD dimerization. Furthermore, crystal structures have been solved for apoenzyme, binary complexes of the enzyme with cofactor or substrate, and the abortive ternary complex formed by HAD, NAD^+^, and acetoacetyl-CoA (AACoA) [21]. These structures illustrated that both the cofactor and the substrate are in the active site. In addition, the optical absorption spectrum of the abortive ternary complex revealed a novel absorption peak that represents the formation of the HAD catalytic intermediate, the charge transfer complex [21]. Interpretation of these data provided a better understanding of the catalytic mechanism as well as the role of key amino acids in the substrate recognition.

**Figure 1:**
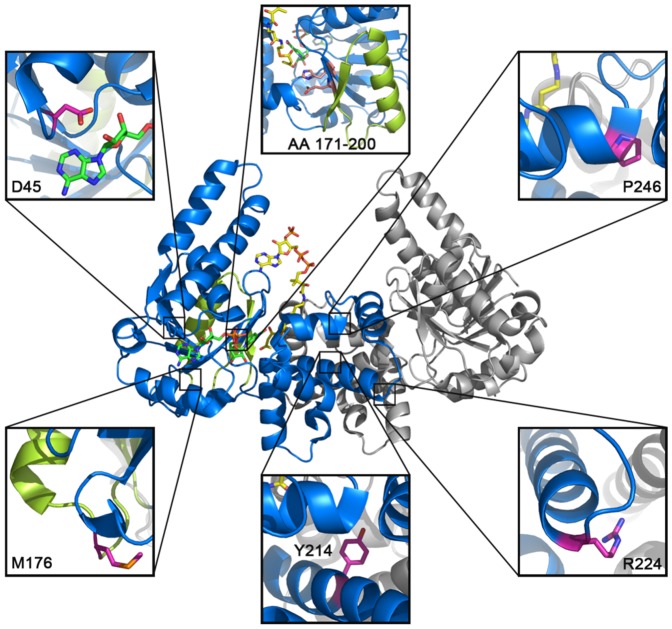
Mapping disease related mutations into human HAD crystal structure (PDB entry 1F0Y). Two monomers of HAD are colored in blue and grey, respectively. The carbon atoms of NAD^+^, acetoacetyl-CoA (AACoA), side chains of mutated residues and catalytic His-Glu pair of one monomer (blue) are colored in green, yellow, magentas and salmon, respectively. Regions colored in lemon refer to the in-frame deletion from 171–200 (c.547-3_549 deletion in *HAD* gene). If not mentioned, all the structure illustrations were generated with *PyMOL* (http://www.pymol.org. Accessed 2014 March 26^th^.).

By mapping the reported disease-relevant HAD mutations onto the crystal structure of human HAD (PDB entry 1F0Y) (**Fig. 1**), we could expect that the mutations involving the active site (c.547-3_549del, i.e. truncation of a.a. 171–200, see also **Table 1** and **Fig. 1**) and the cofactor-binding pocket (D45G and M176V, see also **Table 1** and **Fig. 1**) would decrease enzyme activity by directly affecting the catalytic cavity and the substrate binding. Those identified mutations on C-terminal domain (P246L, IVS6-2a>g, Y214H and R224X) that resides far away from the catalytic center and contributes to HAD dimerization were also proved to be the genetic cause of the diseases (**Table 1** and **Fig. 1**). However, such important correlation between the C-terminal domain and the catalytic activity is yet uncharacterized.

*Caenorhabditis elegans* (*C*. *elegans*) has been used as a model organism for studying the fatty-acid metabolism regulation. In 2003, hundreds of worm fat regulatory genes have been identified using the genome-wide RNA-mediated interference (RNAi) analysis [22]. Recently, 3-hydroxyacyl-CoA dehydrogenase from *C*. *elegans* (*c*HAD, WormBase ID: WBGene00010035) was identified as a novel obesity gene [23], whose amino acid sequence shares 49% identity with human HAD. According to our knowledge, *c*HAD also exists as a dimer in solution as the human HAD does [24]. Here, we investigated the correlation between the dimerization interface and the enzyme activity of *c*HAD in molecular details. We determined the crystal structure of *c*HAD and analyzed its dimerization interface. Three *c*HAD mutations with alterations of the dimerization interface were constructed and their folding, oligomeric states, cofactor-binding behaves, enzymatic activities, ternary complex formation and structural thermal stabilities were examined and compared with those of the wild type *c*HAD. Further molecular dynamics simulations revealed that the alteration of the dimerization interface increases the fluctuation of the distal substrate-binding region, which provides an explanation of how the HAD dimerization mediated by the C-terminal domain affects its catalytic activity.

## Materials and Methods

### Protein production, crystallization and structure determination

The expression, purification, crystallization and data collection processes of *c*HAD were carried out as described previously [24]. In brief, *c*HAD was expressed in *Escherichia coli* strain BL21 (DE3) with a C-terminal GST (Glutathione S-transferase)-tag using the pEXS-CG recombinant vector, a vector developed according to the pET-22b (+) vector (Novagen) in our laboratory. The GST-cleaved protein was purified by affinity chromatography followed by ion-exchange chromatography. Crystals of *c*HAD were grown by the hanging drop method under 23% PEG (polyethylene glycol) 3350, 0.2 *M* sodium chloride, 0.1*M* N,N-Bis (2-hydroxyethyl) glycine pH 8.0. These crystals were soaked in a cryo-protectant consisting of 15% PEG 3350, 0.3 *M* sodium chloride and 20% (*v*/*v*) glycerol and were flash-cooled in nitrogen gas stream at 100 K. Diffraction data were collected at Beamline BL5A of Photon Factory (KEK, Japan) and processed using the *HKL*-2000 package [25].

The structure of *c*HAD was determined by the molecular replacement using Phaser [26] with the human HAD structure (PDB entry 3had) [19] as a search model. The initial model was built using *ARP/wARP* (*CCP*4*i*) followed with an iterative refinement and the model building using *REFMAC5* (*CCP*4*i*) and Coot [27]. Data collection and refinement statistics are summarized in **Table 2**.

**Table 2:** Data collection and refinement statistics*.

	PDB entry 4j0f
Space group [No.]	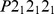 [No. 19]
Unit-cell parameters (Å, °)	*a* = 62.6, *b* = 96.4, *c* = 114.2,
	*α* = *β* = *γ* = 90°
Data-collection statistics	
Wavelength (Å)	1.000
Resolution (Å)	50.00–2.20 (2.24–2.20)
Total reflections	257631
Unique reflections	35658
Redundancy	7.2 (7.1)
Completeness of data (%)	99.9 (100.0)
*I*/*σ* (*I*), overall	20.96 (3.28)
*R*_merge_ † (%)	0.091 (0.366)
Refinement statistics	
Resolution range (Å)	73.66–2.20
*R*_work_ § (%)	19.01
*R*_free_ ¶ (%)	24.39
R.m.s.d bonds (Å)	0.017
R.m.s.d angles (°)	2.003
Average B value (Å^2^)	37.937
Ramachandran plot: main-chain torsion-angle statistics (%)	
Most favored	97.1
Allowed	2.9
Disallowed	0

*The data set was collected from one single crystal. Values in parentheses are for the highest resolution shell.

† R_merge_  = ∑_hkl_∑_I_ |*I*_i_(*hkl*) − *<I*(*hkl*)*>*|/∑_hkl_∑_i_ *I*_i_(*hkl*), where *I*_i_(*hkl*) is the intensity of the *i*th observation of the reflection *hkl* and *<I*(*hkl*)*>* is the mean intensity of reflections *hkl*.

§ R_work_  = ∑(||*F*(*obs*)| − |*F*(*calc*)||)/∑|*F*(*obs*)|, where *F*(*obs*) and *F*(*calc*) are observed and calculated structure factors, respectively.

¶ *R*_free_ was calculated using 5% of data excluded from refinement.

### Site-directed mutagenesis

Site-directed mutagenesis was performed by the overlapping PCR method. The recombinant plasmid of the wild type *c*HAD was used as a template DNA and the PrimeSTAR HS DNA Polymerase (TaKaRa) was used for the DNA amplification. The amplified DNA containing mutations were ligated into the vector pEXS-CG at the restriction site by *Bam*HI and *Eco*RI, and were verified by sequencing. The mutant *c*HAD proteins were expressed and purified under the same procedure as the wild type.

### Size-exclusion chromatography coupled with in-line static light-scattering, refractive-index and ultraviolet measurements (SEC-LS/UV/RI)

We used size-exclusion chromatography measurements to determine the oligomeric state of the wild type and mutant *c*HADs in solution by a method that Li et al described previously [28]. Briefly, the SEC-LS/UV/RI instrumental setup consists of an Agilent 1100 HPLC system (Agilent Technologies) that is connected in series to a DAWN HELEOS II light-scattering detector (Wyatt Technology) and an Optilab rEX interferometric refractometer detector (Wyatt Technology). And this system can simultaneously monitor ultraviolet absorption (UV), light scattering (LS) and refractive index (RI) respectively. Before the sample injection, a WTC-030S5 column (Wyatt Technology) was equilibrated with a mobile phase consisting of 20 m*M* 2-(N-morpholine)-ethane sulphonic acid pH 6.0, 100 m*M* sodium chloride. 100 µg of purified protein was injected onto the column and eluted at a flow rate of 0.5 ml/min. The column effluent was monitored in-line with three detectors mentioned above. After correcting the inter-detector volume delays between detectors, the three resulting chromatograms were aligned by the *ASTRA V* software (Wyatt Technology) and used for calculating the oligomeric molecular weight of sample protein.

### Chemical cross-linking assay

Ethylene glycolbis (EGS, Thermo Scientific) was dissolved in Dimethyl sulfoxide (DMSO, Sigma-Aldrich) to yield the EGS stock solution with the concentration of 22 m*M*. The protein samples were diluted to 2 mg/ml in the buffer containing 0.2 *M* Na_2_HPO_4_ and 0.1 *M* citrate, pH 7.0. All reagents were precooled on ice for 30 min before the cross-linking reaction. Then 10 µl of the protein solution and 1.3 µl of the EGS solution were mixed to reach a final reaction volume of 20 µl by adding 8.7 µl water. The molar ratio between the protein and the EGS was 1∶50. The cross-linking reaction was carried out on ice. After 40 min, the reaction system was mixed with 5 µl SDS-PAGE loading buffer (5X) to terminate the reaction, and then subjected into SDS-PAGE analysis.

### Internal tryptophan fluorescence by NADH titration

There is one residue Trp proximal to the NADH binding pocket and the distance allows the FRET (fluorescence resonance energy transfer) effect between the residue and the bound NADH. Therefore, we measured the internal tryptophan fluorescence changes by NADH titration to determine the cooperation effect of NADH binding by the enzyme. Fluorescence measurements were performed on Varioskan Flash spectrofluorimeter (Thermo) at the ambient temperature. The excitation wavelength was set to 270 nm and the emission spectra were scanned from 290 to 500 nm. Enzyme was incubated with a different concentration of NADH in 10 m*M* citrate/phosphate buffer, pH 7.0, 150 m*M* sodium chloride in a total reaction volume of 100 µl. The concentration of the protein was kept to 0.58 µ*M* and the concentration of NADH was varied from 0 to 340 µ*M*. The fluorescence intensity of NADH at 460 nm was designated as *F*. The minimum and maximum fluorescence intensity corresponding to 0 and 340 µ*M* NADH concentration was designated as *F*_min_ and *F*_max_, respectively. To obtain the number of NADH binding site, data was fitted to the logarithmic Hill equation:
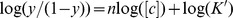



Here *y* was calculated from (*F*–*F*_min_)/(*F*_max_–*F*_min_), [*c*] is the concentration of NADH, *K*′ is a constant comprising of interaction factors and dissociation constant and the value of *n* is equivalent to the number of binding site within 10–90% saturation of the active site [29].

### Enzymatic activity assay

Enzymatic activity of *c*HAD was measured on a Hitachi U2010 spectrophotometer at 298 K by monitoring the decrease of NADH concentration at 340 nm upon reduction of acetoacetyl-CoA (AACoA) as described by Noyes and Bradshaw [30]. All reactions were performed in a 0.1 *M* citrate/phosphate buffer, pH 7.0, 0.1 m*M* dithiothreitol with the protein concentration kept to 23 n*M* in a total reaction volume of 500 µl. The initial rates were determined in triplicate at a saturating NADH concentration of 100 µ*M* and varied AACoA concentration from 30 to 70 µ*M*. Data were then fit to the Michaelis-Menten equation to obtain V_max_ and *K*_m_. Kinetic parameters are summarized in **Table 3**.

**Table 3:** Kinetic parameters of wild type and mutant *c*HAD enzymes.

	*K*_m_ (µ*M* acetoacetyl-CoA)	V_max_ (nmol/s/µg enzyme)	*k*_cat_ (s^−1^)	*k*_cat_/*K*_m_ (µ*M* ^−1^s^−1^)	*n* a
Wildtype	72.0±13.4	1.3±0.1	45	0.6	1.1±0.04^a^
R204A	83.2±9.7	0.8±0.05	28	0.3	1.2±0.04^a^
Y209A	50.4±11.0	0.5±0.06	17	0.3	1.1±0.03^a^
R204A/Y209A	115.6±24.5	0.3±0.04	10	0.09	1.2±0.02^a^

a Hill coefficent and the values are measured from fluorescence data.

### Difference spectroscopy of *c*HAD·NAD^+^·AACoA ternary complex

The formation of *c*HAD·NAD^+^·AACoA ternary complex was monitored by the difference spectroscopy. Spectroscopic measurements were performed on Varioskan Flash spectrofluorimeter (Thermo) at ambient temperature. 0.2 *M* citrate/phosphate buffer, pH 7.0 was used as the buffer system and the final volume was always 100 µl. Aliquots of stock solutions were added to attain ligand concentrations of 2 m*M* (NAD^+^ and/or AACoA) and a protein concentration of 2 mg/ml. Spectra were collected from 350 to 600 nm and the difference spectra were calculated by subtracting the sum of the AACoA spectrum and *c*HAD·NAD^+^ spectrum from the spectrum of the ternary complex. Measurements were made in triplicate. Results were calculated and plotted by Origin 8.0 software package.

### Protein thermal stability by thermal shift assay

The protein samples were diluted to 1.5 mg/ml in the buffer containing 0.2 *M* Na_2_HPO_4_ and 0.1 *M* citrate, pH 7.0. The fluorescent dye SYPRO Orange (Invitrogen) was added into the sample solution by ∼1,000 fold of dilution. 20 µl of mixture in a PCR tube was heated up from 25°C to 75°C with the step of 1°C per min according to the reported protocol [31], [32]. The fluorescence of the mixture was measured by using a RT-PCR device (Corbett 6600). The melting temperature (*T*_m_) was estimated as the temperature corresponding to the minimum of the first derivative of the protein denaturation curve. All the measurements were repeated three times.

### Circular dichroism (CD) spectroscopy

Purified protein samples were diluted to 0.2 mg/ml in the phosphate buffer that was made by dissolving 0.24 g KH_2_PO_4_, 1.44 g Na_2_HPO_4_, 0.2 g KCl and 8 g NaCl into 1-liter water. The diluted samples were used to measure their CD spectra respectively. The spectra were recorded over the wavelength from 200 nm to 260 nm with a bandwidth of 1 nm and 0.5 s per step by using CD spectrometer (Chirascan-plus, Applied photphysics). All the measurements were repeated three times and the spectrum data were corrected by subtracting the buffer control.

### Molecular dynamics simulation

The MD simulations were performed using NAMD 2.9 [33] and the CHARM 22/27 force field with CMAP correction [34]. Simulations were performed under constant NPT conditions, and periodic boundary conditions are applied. The temperature was maintained throughout at 310 K using a Langevin thermostat with 5 ps^−1^ damping coefficients. The system pressure was maintained at 1 atm using a Langevin piston barostat. Electrostatic interactions were calculated using the particle mesh Ewald sum method [35] with a cutoff of 12 Å. An integration time step of 2 fs was used while constraining all hydrogen-containing covalent bonds with the SHAKE algorithm [36].

Three simulation systems were constructed for the wide type, R204A and R204A/Y204A *c*HAD dimer, respectively. Solvation, ionization, minimization and MD equilibrium protocols were carried out as follows. Each simulation system was solvated with TIP3P water, and neutralized by K^+^ and Cl^−^ counter ions. Then each system was equilibrated using a stepwise relaxation procedure. Initially, the system was energy minimized for 20 ps with non-hydrogen atoms of protein and crystal water molecules fixed. The system was then heated from 0 K to 310 K stepwise with 10 K raise per 2 ps while harmonically restraining (with spring constant 5 kcal.mol^−1^.A^−2^) the protein backbone and oxygen atoms of crystal water molecules. The system was pre-equilibrated in the canonical ensemble with the same harmonic restraints for 1 ns. Constraints were next released stepwise with duration 40 ps for each step. The spring constant decreases to 4, 2 and 1 with no additional atoms released. As following, only C-alpha atoms of the protein were constrained and the spring constant decreases to 1, 0.2 and 0.05. After all restraints on C-alpha of the protein atoms were removed, regular NPT simulations were executed. A total of 200, 212 and 218 ns of simulation data were generated for the wide-type, R204A and R204/Y204A *c*HAD dimers, respectively.

## Results and Discussions

### Overall structure and dimerization interface of *c*HAD

**Figure 2:**
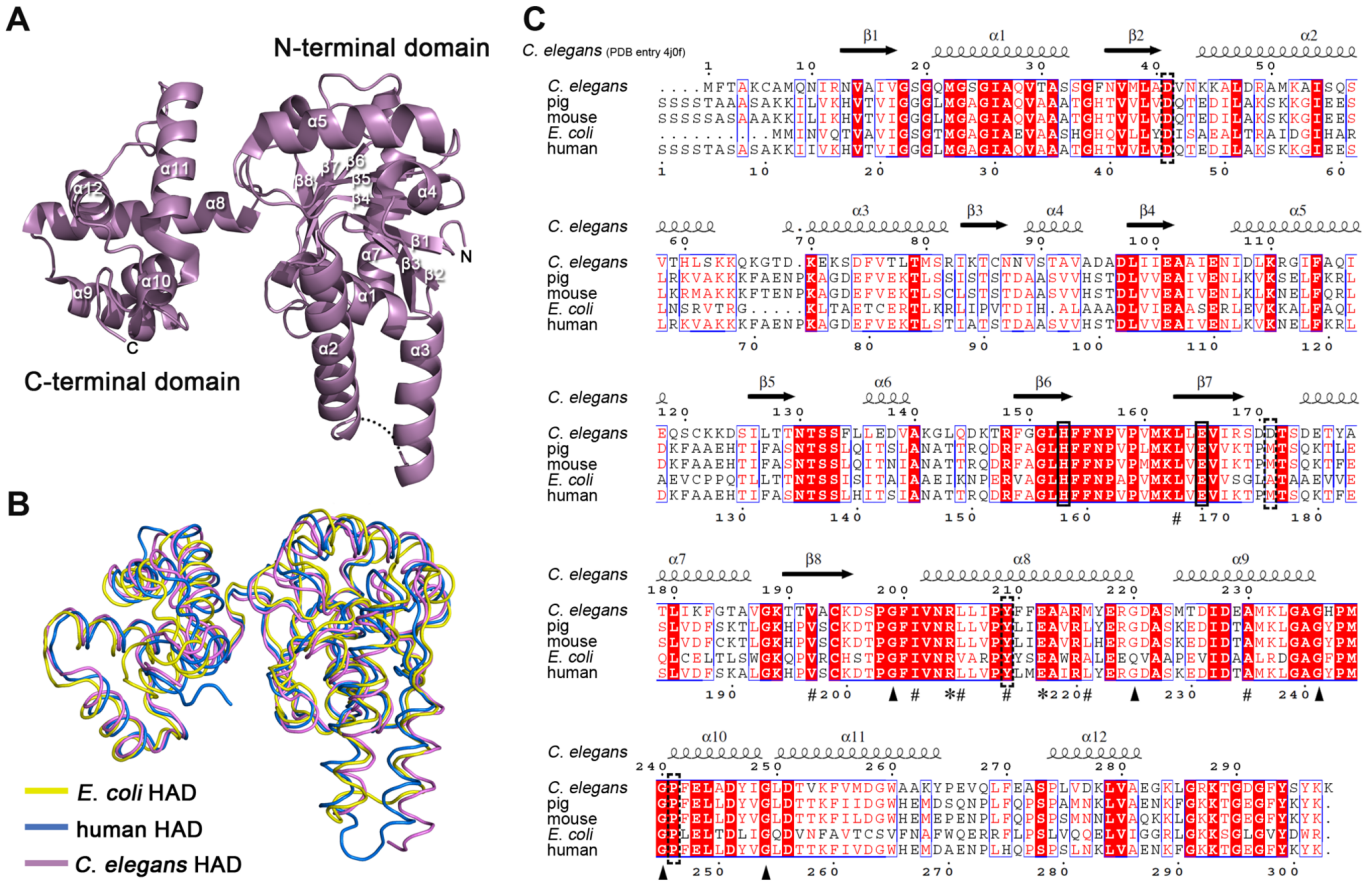
Overall structure of *c*HAD and protein primary structure comparison between *c*HAD and its homologues. (**A**) Ribbon diagram of *c*HAD crystal structure (PDB entry 4J0F, this work) colored in purple. The loop between helix α2 and α3 with weak electron density is indicated by dotted line. (**B**) Tertiary structure alignment among 3-hydroxyacyl-CoA dehydrogenases from *C*. elegans (purple, PDB entry 4J0F), human (blue, 3HAD) and *E*. *coli* (yellow, 3MOG). The region from residue 286 to 475 in *E*. *coli* HAD is not shown. (**C**) Sequence alignment among 3-hydroxyacyl-CoA dehydrogenases from *Caenorhabditis elegans* (*C*. *elegans*, GenBank accession No. CAA80153.1), *Homo sapiens* (human, GenBank accession No. CAA65528.1), *Sus scrofa* (pig, GenBank accession No. AAD20939.1), *Mus musculus* (mouse, GenBank accession No. BAA06122) and *Escherichia coli* (*E*. *coli*, GenBank accession No.NP_415913.1, residues 1–286). The transit peptide sequence was excluded from human, pig and mouse HAD sequences. The secondary structures are corresponding to *c*HAD. The catalytic His-Glu pair is boxed in black. The disease related point mutations in **Table 1** are boxed with dashed line. The conserved glycine residues flanking C-terminal domain helices are indicated by triangles. The conserved Arg and Glu forming salt-bridge on dimerization interface are indicated by asterisks; and the conserved hydrophobic residues on the dimerization interface are indicated by “#”. The labels of secondary structure are corresponding to those in (**A**).

The crystal structure of *c*HAD was refined to 2.2 Å in space group *P*2_1_2_1_2_1_ with the final R_work_ of 19.0% and R_free_ of 24.4%, respectively (**Table 2** **and Fig. S1**). The overall fold of *c*HAD monomer exhibits a two-domain topology similar to the crystal structure of the human HAD (**Fig. 2A, 2B**). The N-terminal domain mainly involving cofactor binding (residues 9–194) consists of a core eight-stranded *β*-sheet flanked by α-helices. The first six stands of the sheet arrange in a conformation as observed in a typical Rossmann fold. The final two stands are also parallel but run in the opposite direction against β1–β6. An interesting feature of the N-terminal domain is the helix-turn-helix protrusion formed by helix α2 and α3. The C-terminal domain (residues 202–297) contains a bundle of five α-helices (α8-α11) and is connected to the N-terminal domain by a flexible loop (residues 195–201). Interestingly, from the sequence alignment, the C-terminal domain of HAD is more conserved than the N-terminal domain, implying the important role of HAD C-terminal domain for its biological function (**Fig. 2C**). Furthermore, a couple of conserved glycine residues (residue 199, 220, 236, 240, 249, **Fig. 2C**, indicated by triangles) flanking C-terminal *α*-helices yield the flexibility of these helices to adjust their relative orientation to one another.

**Figure 3:**
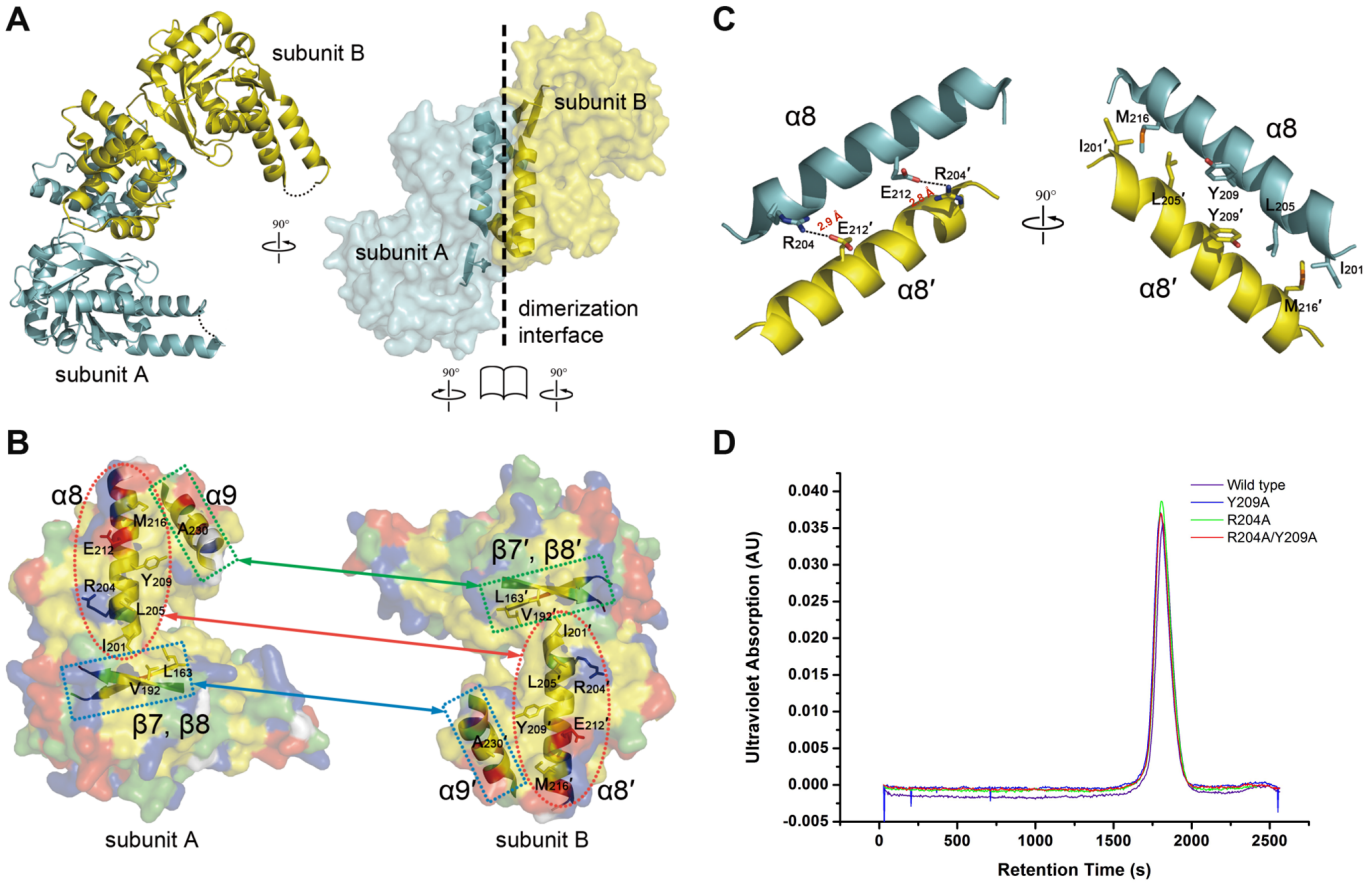
Dimerization interface of *c*HAD. (**A**) Ribbon (left) and surface (right) diagram of the crystal structure of *c*HAD dimer in an asymmetric unit. The two molecules, subunit A and subunit B, colored in cyan and yellow respectively in the ribbon diagram, are arranged in a “tail-to-tail” manner through interactions between their C-terminal domains. The loop between helix α2 and α3 with weak electron density is indicated by dotted line. In the surface diagram, only secondary structures involving dimerization are depicted. (**B**) An “open-book” view of the dimerization interface between subunit A and B. The negatively charged, positively charged, polar, hydrophobic and glycine residues on the surface are represented in red, blue, green, yellow and white, respectively. The contact sites of the dimerization interface via α8/α8′, α9/β7′–β8′ and α9′/β7–β8 are indicated by the red dotted ellipse, green dotted rectangle and blue dotted rectangle. (**C**) Core dimerization interface. A combined ribbon and stick model illustrates both electrostatic (left) and hydrophobic (right) interactions between each α8 helix of two subunits. Salt bridges are indicated with the dashed lines. (**D**) Gel filtration profile of the wild type and mutated *c*HADs. Equal amount of protein (100 µg) was injected onto a pre-equilibrated Superdex 200 column (10/300 GL; GE healthcare) and eluted at a flow rate of 0.5 ml/min.

Within one asymmetric unit, two *c*HAD molecules mutually contact through their C-terminal domain and form a dimer (**Fig. 3A**). The dimerization of two subunits is mediated primarily by hydrophobic interactions. The core hydrophobic interactions are contributed from the helix α8 (residues 201–219) of each subunit (**Fig. 3A**). In addition, the hydrophobic interactions between stands β7 and β8 of the N-terminal domain on one subunit and the helix α9 (residue 224–235) on another subunit further stabilize the subunit dimerization (**Fig. 3B**). The hydrophobic residues involving dimerization are evolutionally conserved across species according to sequences alignment among HAD homologues (**Fig. 2C**, indicated by “#”; **Fig. 3C**, right). Besides hydrophobic interactions, the distinctive antiparallel arrangement of α8 from two subunits is also anchored by two pairs of salt bridges formed between R204 on one subunit and E212 on the opposing one (**Fig. 3C**, left). And this Arg/Glu residue pair is also highly conserved among different species (**Fig. 2C**, indicated by asterisks).

To further evaluate the above dimerization interface that is not simply from crystal packing contact, we render our *c*HAD crystal structure to the web server PDBePISA [37], [38] for protein interfaces analysis. As expected, the interface between two α8 helices gained a P-value of 0.025 and scored 1.0, which implies that this interface plays an essential role in complex formation, instead of being a result of crystal packing. The solvent-accessible area of the dimerization interface reaches 1640 Å^2^, as much as 11% of total area of dimer, 14400 Å^2^, while number of residues participating in interface formation occupies 15% of total 289 residues. As a result, the interface mediated by the α8 helices is indeed the core dimerization interface of *c*HAD.

### Attenuating interactions within the dimerization interface of *c*HAD

According to the above structural analysis of *c*HAD dimerization interface, we performed mutagenesis with the aim of attenuating interactions on the dimerization interface (**Fig. 3C**). Three mutants (R204A, Y209A and R204A/Y209A) with the mutations on the core α8 dimerization interface were constructed. R204A was designed to attenuate the electrostatic interaction between helices. Y209A was designed to weaken the hydrophobic interaction on the dimerization interface. R204A/Y209A is a combination of attenuation of both electrostatic and hydrophobic interactions. With the same expression and purification procedure, the protein yields of mutant *c*HADs were approximately 5-fold lower than that of wild type *c*HAD. For the mutants (e.g. R204E) by changing the interface interactions severely, the protein was expressed and appeared as inclusion body (data not shown), which suggests that blocking the dimerization interface of *c*HAD will affect the protein folding and stability.

The molecular weight of wild type *c*HAD oligomer in solution was determined using the SEC-LS/UV/RI experiment (**Fig. S2A**; see also Materials and Methods). In consistency with the dimeric state observed in the crystal structure, the molecular weight of wild type *c*HAD oligomer in solution was 69.7 kDa, twice as its subunit molecular mass of 35 kDa. This result confirms that *c*HAD assembles into a dimer in solution. In addition, the molecular weight of R204A, Y209A, R204A/Y209A mutant *c*HADs was determined as 69.1, 71.1 and 70.6 kDa, respectively (**Fig. S2A**), which are close to that of the wild type *c*HAD oligomer.

Another gel-filtration experiment by loading an equal amount of protein exhibited a single symmetric elution peak for both the wild type and mutant *c*HADs (**Fig. 3D**), suggesting that monomer of *c*HAD does not exist and thereby there is no equilibrium between the monomer and the dimer for all *c*HADs. The equal elution peak heights also reveal the similar solubility and stability for all *c*HADs.

We further investigated the dimerization behavior of *c*HAD using the chemical cross-linking approach (**Fig. S2B**). There is no significant difference for the ratio of monomer and crossed-linker dimer among the wild type and mutant *c*HADs, implying that the conformations of all *c*HAD dimers are similar in the solution.

Therefore from all above, the alteration of the dimerization interface from the three mutants (R204A, Y209A and R204A/Y209A) would decrease the subunit interaction but does not affect the final dimerization state in the solution, which enabled us to further investigate how the altered interface influences the enzymatic activity of *c*HAD.

### Enzymatic activities of *c*HAD and its variants

In comparison to the wild type, the mutants R204A, Y209A and R204A/Y209A have their V_max_ values significantly reduced to 62%, 38% and 23%, respectively (**Table 3**). Besides, all three mutants exhibit comparable *K*_m_ values with the wild type. Therefore, attenuating interactions on the dimerization interface distinctly impair the enzyme catalysis efficiency more significantly than affecting their substrate binding affinity. R204A/Y209A mutation shows the most evidently impaired enzymatic activity based on its *k*_cat_/*K*_m_ value, which reduced approximately 10-fold of that of wild type *c*HAD. The enzymatic activity of the two single site mutations, R204A and Y209A, decreased to half of the wild type.

Among the disease-related human HAD mutations on the C-terminal domain, the mutation site of Y214 located on the central dimerization interface (**Fig. 1**) corresponds to Y209 in *c*HAD. The over expressed mutant enzyme Y214H has no detectable activity *in vitro* [16]. It should be noted that the mutation of Y214H would have more severe effects on the dimerization interface than the mutation of Y209A that just attenuates the interaction of the dimerization interface.

Therefore, attenuating the interaction of the dimerization interface by mutating R204 and Y209 to Ala does not change the dimeric state but significantly weaken the enzymatic activities of all three mutants.

### Negative cooperation effect of NADH binding within the *c*HAD dimer

The dimerization interface of *c*HAD in the C-terminal domain is away from the substrate-binding site and the catalytic pocket in the N-terminal domain. To understand the distal effect of the dimerization interface in the enzymatic activity regulation, we raised one hypothesis that the cooperation effect exists between the two subunits within the dimer during the catalytic process, i.e. the two subunits within the dimer could sense each other via the dimerization interface and catalyze the reaction in a synergetic way.

**Figure 4:**
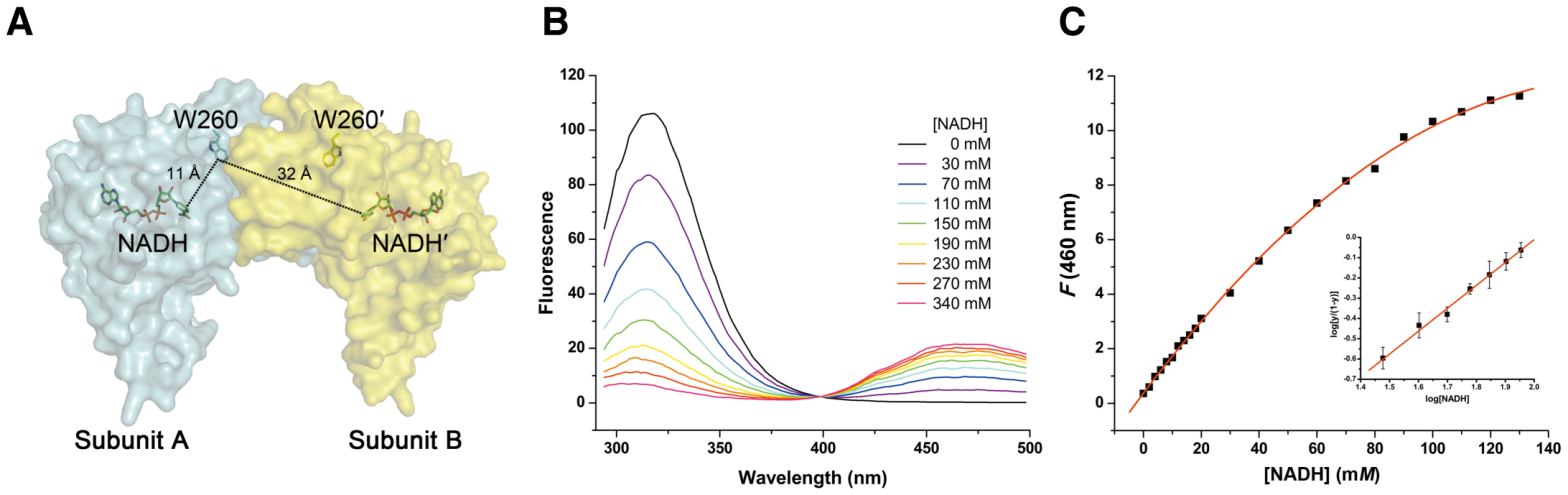
Negative cooperation effect of NADH binding within the *c*HAD dimer. (**A**) A combined surface and stick model presents the distances between the side chain of intrinsic W260 and bound NADHs of subunit A (cyan) and subunit B (yellow). The positions of NADHs are determined by superposition of the crystal structure of human HAD·NADH complex (PDB entry 1F17) with the present *c*HAD crystal structure. (**B**) Fluorescence resonance energy transfer (FRET) spectrum by titrating NADH with the wild type *c*HAD. The excitation wavelength is 270 nm and the emission wavelength is scanned from 290 to 500 nm. The FRET signal appears at the wavelength of 460 nm. (**C**) Hyperbolic curve of the FRET fluorescence signal at 460 nm against the titration of NADH concentration for the wild type *c*HAD. The insert represents the logarithmic Hill plot between 10 and 90% active site saturation.

To assess the above hypothesis, we utilized the fluorescence resonance energy transfer (FRET) approach to study the potential cooperation effect of NADH binding within the dimer. We found a significant FRET signal between the bound cofactor NADH and the only intrinsic tryptophan residue W260 located at helix α11 (**Fig. 4A**). The distance between W260 and NADH on the same subunit or NADH on the opposite one is 11 and 32 Å, respectively (**Fig. 4A**), both of which are within the Förster distance [39] and could sustain the occurrence of FRET phenomenon. As a result, the bound NADH could absorb the fluorescence (340 nm) of W260 that is excited by the light of 270 nm and emit the FRET fluorescence at 460 nm. However, the efficiency of this energy transfer is inversely proportional to the sixth power of the distance between donor and acceptor due to the dipole-dipole coupling mechanism [40]. Hence, the FRET efficiency between W260 and NADH on the opposite subunit is approximately 1000 times weaker than the FRET efficiency between W260 and NADH on the same subunit. Therefore, the overall FRET phenomenon of a dimer could be considered as the sum of FRET phenomenon within each monomer.

When we titrated the protein by adding NADH with an increased concentration, a clear FRET signal of NADH fluorescence emission at 460 nm could be observed (**Fig. 4B**). Hyperbolic curve of the dependence of fluorescence emission on NADH concentration excludes the possibility of positive cooperative binding of NADH (**Fig. 4C**). By calculating the number of NADH binding site, it is shown that NADH binding is negatively cooperative with the Hill coefficient *n*∼1 (**Table 3**), which indicates that only one NADH molecule can bind to the dimeric enzyme every time.

Surprisingly, the above mutations on the *c*HAD dimerization interface did not change the NADH binding cooperative effect distinctly (**Table 3**). Wild type and Y209A mutant *c*HAD show exactly the same number of Hill coefficient *n* = 1.1 while the mutants R204A and R204A/Y209A have a slightly increased *n* of 1.2, possibly implying a relatively weakened negative synergetic effect of NADH binding in these two mutants.

Although NADH titration experiments did not address the effect of HAD dimerization interface alteration on its enzymatic activity, our data here on the cofactor binding cooperation effect of *c*HAD does disagree with the previous report that there are two NADH molecules in the crystal structure of HAD·NADH binary complex [21]. A possible explanation would be that, in their experimental procedures, the HAD·NADH complex was obtained by soaking the apo-enzyme crystals with a high concentration of NADH. The NADH molecule could bind randomly to either HAD subunit of the dimer. As a result, the crystallographic averaging yields electron densities of NADH equally at both cofactor binding sites of HAD. The averaged B-factors (48 Å^2^ and 60 Å^2^) of two NADH cofactors in the dimer are obviously higher than the averaged one (41 Å^2^) of the whole protein (PDB entry 1F17) [21], supporting the above explanation.

To further understand the negative cooperation effect of NADH binding by cHAD dimer, the wild type protein and two mutants R204A and R204A/Y209A are selected to perform molecular dynamics simulations (**Fig. S3**). All three simulations reached equilibrium at about 50 ns and continued till about 200 ns (**Fig. S3A**). The time evolution of the RMSD (root-mean-square deviation) between two subunits within the dimer was plotted in **Fig. S3B**. The RMSD of subunit one with respect to subunit two is ∼2 Å for the wild type and ∼3 Å for the mutants, implying two subunits behave asymmetrically. Such an asymmetric behavior is in line with the above experimental observation that only one NADH could bind to the *c*HAD dimer every time (**Fig. 4C** and **Table 3**). We speculate here that the structural RMSD could vibrate if a longer simulation can be executed.

### Alteration of dimerization interface affects the formation efficiency of HAD catalytic intermediate

The above NADH titration assay shows that alterations of the dimerization interface by the three mutants do not affect the negative cooperation effect of cofactor binding. We therefore sought to further evaluate the role of the dimerization interface in HAD catalysis. We utilized the difference absorption spectroscopy approach to investigate the formation of the charge transfer complex intermediate of *c*HADs. It has been reported that the abortive ternary complex comprising HAD, NAD^+^ and AACoA exhibits a broad absorbance band centered between 410 and 420 nm. This unique band is not appreciable in individual spectra of HAD, NAD^+^, AACoA or the binary combinations of HAD·NAD^+^, HAD·AACoA, and NAD^+^·AACoA [21]. The spectroscopic properties of the charge transfer complex are sensitive to perturbations in the protein structure and can been used to probe the integrity of the active site.

**Figure 5:**
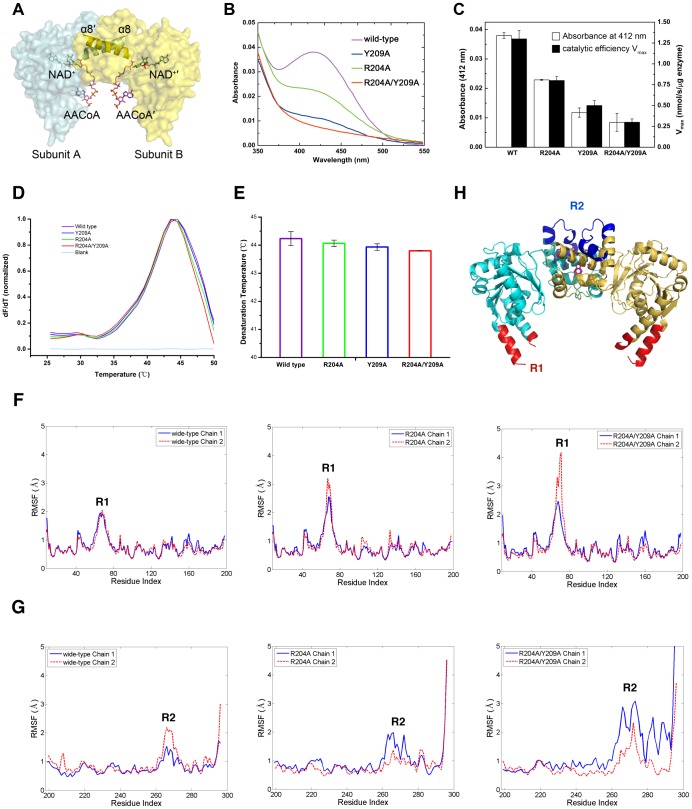
Characterization of the charge transfer complex intermediate formation. (**A**) A combined surface and stick model presents a structural model of *c*HAD ternary complex with the NAD^+^ and AACoA bound. The α8 helices of the dimer are depicted as ribbons. The positions of NAD^+^ and AACoA are determined by superposition of the structure of human HAD·NAD·AACoA complex (PDB entry 1F0Y) with the present *c*HAD crystal structure. (**B**) Difference absorption spectra of the *c*HAD·NAD^+^·AACoA complexes for the wild type (purple), R204A mutant (green), Y209A mutant (blue) and R204A/Y209A mutant (red) at a ligand concentration of 2 m*M* as described in **MATERIALS AND METHODS**. (**C**) Effects of mutations on charge transfer complex formation. The columns filled in white represent the net absorbance of ternary complex formed by *c*HAD and its variants at 412 nm (scaled by left vertical axis), while the columns filled in black represent their V_max_ values determined in kinetic experiments (scaled by right vertical axis). (**D**) Thermo shift assay of *c*HAD and its variants. (**E**) The critical melting temperature (*T*_m_) of *c*HAD and its variants from the thermo shift assay in (D). (**F**) The root-mean-square fluctuations (RMSF) of *c*HAD (wild type at left, R204A at middle and R204A/Y209A at right) N-terminal domain residues (1–198) for each subunit (chain 1 and 2). The significant fluctuation regions (R1, a.a. 60–80) are label accordingly. (**G**) The RMSFs of *c*HAD (wild type at left, R204A at middle and R204A/Y209A at right) C-terminal domain residues (199–297) for each subunit (chain 1 and 2). The significant fluctuation regions (R2, a.a. 260–280) are label accordingly. (**H**) Cartoon representation of the crystal structure of *c*HAD with one subunit colored in cyan and another in gold. The significant fluctuation regions R1 (a.a. 60–80) and R2 (a.a. 260–280) observed in molecular dynamics simulation (F and G) are colored in red and blue, respectively.

The superposition between our *c*HAD crystal structure and previous reported abortive ternary complex of human HAD [21] enabled us to build a model of abortive ternary complex of *c*HAD (**Fig. 5A**). The core dimerization interface α8 does not contribute to either the substrate/cofactor binding site or the catalytic active site. Alteration of the core dimerization interface, apparently, does not affect the formation of the charge transfer complex intermediate. However, the difference spectroscopy of *c*HAD·NAD^+^·AACoA ternary complex (**Fig. 5B**) showed that the absorption spectrums between 410 and 450 nm were significantly reduced for the three mutants (R204A, Y209A, R204A/Y209A) in comparison to that of the wild type enzyme. This indicates that the formation efficiency of the charge transfer complex decreases for the mutants. The absorbance at 412 nm of R204A, Y209A and R204A/Y209A mutants reduced to 60%, 31% and 22% of that of the wild type, respectively. Interestingly, such absorbance reductions of mutants exhibit high concordance with the decrease of their catalytic efficiency V_max_ (**Fig. 5C**), which strongly indicates that the reduced enzymatic activity by attenuating the interactions within the dimerization interface is directly relevant to the attenuated efficiency of the catalytic intermediate formation.

CD spectra of *c*HADs show that the mutated proteins preserve the same secondary structures with the wild type (**Fig. S4**). The thermal shift stability experiments reveal that the mutations at the dimerization interface do not significantly affect the thermal stabilities of *c*HADs with the melting temperatures of 44.2°C for the wild type, 44.1°C for R204A, 43.9°C for Y209A and 43.8°C for R204A/Y209A (**Fig. 5D and 5E**). Thus, the attenuated efficiency of the catalytic intermediate formation by the mutations is not due to the protein misfolding and instability. The above gel-filtration (**Fig. 3D** **and Fig. S2A**) and cross-linking data (**Fig. S2B**) also show that the attenuation is not relevant to the dimer dissociation.

We next sought to utilize molecular dynamics simulations to understand the relation between the dimerization interface alteration and the catalytic intermediate formation. Inspecting the 200 ns simulations of three *c*HADs (wild type, R204A and R204A/Y209A), we found that the mutants exhibit a reduced distance between the helix α8 and α8′ by measuring the C_α_ distances between the pairs of interacting residues (**Table 4** **, Fig. S3C and S3D**) and such reduced distance allows the helix α8 and α8′ to form a closer contact to compensate the space by the side chain truncation from Arg/Tyr to Ala.

**Table 4:** Averaged C_α_ distances of corresponding residues from molecular dynamics simulations^a^.

	R204 - E212′ (Å)	R204′ - E212 (Å)	L205 - Y209′ (Å)	L205′ - Y209 (Å)
Wild type	8.74±0.40	8.75±0.32	6.95±0.39	6.90±0.38
R204A	8.53±0.44	8.54±0.55	6.46±0.39	5.55±0.29
R204A/Y209A	7.66±0.48	7.14±0.46	5.13±0.43	5.18±0.37

a The two values represent the averaged distances of C_α_ atoms between corresponding residues and the standard deviation of distances.

Besides the closer contact at the dimerization interface, we investigated the structural fluctuation of *c*HAD as reflected by the root mean square fluctuation (RMSF) of the proteins. With reference to the protein conformation at 50 ns, in the following 150 ns simulations, *c*HAD exhibits large conformational fluctuation around two regions (**Fig. 5F and 5G**), one distal region R1 (a.a. 60–80) and one proximal region R2 (a.a. 260–280) with respect to the dimerization interface. The distal region R1 is located at the helices α2 and α3 of the N-terminal domain and the proximal region R2 is located at the helices α11 and α12 of the C-terminal domain (**Fig. 5H**; see also **Fig. 2A and 2C**). The fluctuations of both regions R1 and R2 increase in the mutant R204A and even more significantly in the mutant R204A/Y209A (**Fig. 5F and 5G**). Thus the alterations of the dimerization interface of *c*HAD could induce overall allosteric effects by affecting the conformational fluctuations of the distal region. We noted that the distal region R1 is involved in the substrate binding according to the model of the ternary complex of *c*HAD (**Fig. 5A**). In the crystal structure of the human HAD ternary complex, the corresponding distal region R1 uses its Lys68 to position the 3′-phosphate of coenzyme A [21] (see also **Fig. S5**). The increased fluctuations of the distal region R1 for the mutants will decrease the substrate binding stability and thereby eliminate the efficiency of the charge transfer intermediate formation.

As a result, our molecular dynamics simulations reveal that the alteration of the dimerization interface of *c*HAD not only affects the interface conformation itself (**Fig. S3C and S3D**) but also modulates the conformational fluctuations of distal regions via a global allosteric effect (**Fig. 5F, 5G and 5H**) and such allosteric effect further eliminates the stability of the catalytic intermediate, thereby weakening the experimental enzymatic activity (**Table 3** **,** **Fig. 5B and 5C**).

### Conclusions

In the current study, we solved the crystal structure of 3-hydroxyacyl-CoA dehydrogenase of *Caenorhabditis elegans* and identified its core dimerization interface. Three mutations on that interface were designed to attenuate the dimerization interactions. All the mutants exhibited similar oligomeric state and negative NADH-binding synergetic effect as compared to the wild type protein. However, the enzymatic activities of the mutants distinctly decreased. Such a decreased activity was in line with the reduced formation efficiency of the charge transfer intermediate complex by difference spectroscopy. Further molecular dynamics simulations showed that the integral dimerization interface interactions are essential for the stability of the catalytic intermediate. Thus the weakened enzymatic activity of *c*HAD by the mutation at the dimerization interface is due to the reduced stability of the catalytic intermediate via an allosteric effect.

It is known that most of allosteric enzymes are oligomeric, and their oligomeric states are evolutionary conserved. Oligomerization is generally thought to be essential for the enzymatic activity regulation. Thus, our studies of *c*HAD provide additional insights into the role of subunit oligomerization in catalysis by tuning the catalytic intermediate formation.

## Accession Code

The atomic coordinates and the structure factors file for the crystal structure of *c*HAD are deposited in the Protein Data Bank with the accession numbers 4J0F.

## Supporting Information

Figure S1 **A stereo image of overall 2Fo-Fc electron density map of** ***c*** **HAD dimer contoured at 1.2 σ.**
(TIF)

Figure S2 **Evaluations of** ***c*** **HAD dimerization state in solution.** (**A**) Molecular weight of oligomeric *c*HAD and its mutants. Aligned SEC-LS/UV/RI differential refractive index chromatograms (right axis) of wild type, R204A, Y209A and R204A/Y209A *c*HADs are represented in purple, green, blue and red dots, respectively. The molecular weights calculated according to LS and RI measurements at each time point are plotted with the scale at the left axis. (**B**) SDS-PAGE analysis of the EGS (Ethylene glycolbis) cross-linked *c*HAD and mutants.
(TIF)

Figure S3 **Molecular dynamics simulations of** ***c*** **HAD.** (**A**) The time evolution of root-mean-square displacements (RMSD) of *c*HAD dimer. Curves for the wide-type, R204A and R204A/Y204A mutants are in purple, green and red, respectively. (**B**) The time evolution plots of RMSD of two subunits (chain 1 and chain 2) within the *c*HAD dimer. (**C**) The time evolution plots of the distances of Cα atoms of residue 204 (in chain 1/2) and residue 212 (in chain 2/1) for the wild type *c*HAD dimer (left) and its mutants R204A (middel) and R204A/Y209A (right). (**D**) The time evolution plots of the distances of Cα atoms of residue 205 (in chain 1/2) and residue 209 (in chain 2/1) for the wild type *c*HAD dimer (left) and its mutants R204A (middel) and R204A/Y209A (right). See also **Fig. 3C** and **Table 4**.
(TIF)

Figure S4 **Circular dichroism (CD) spectra of the wild type and mutated** ***c*** **HADs.** All the measurements were repeated three times and the spectrum data were corrected by subtracting the buffer control.
(TIF)

Figure S5 **Cartoon representation of the crystal structure of human HAD ternary complex (PDB entry 1F0Y).** One subunit is colored in cyan and another in gold. The regions corresponding to the regions R1 and R2 in *c*HAD are colored in red and blue, respectively. The cofactor NAD, substrate AACoA and the residues involved in substrate and cofactor binding are shown in stick model. This figure was prepared using UCSF Chimera (http://www.cgl.ucsf.edu/chimera/. Accessed at 2014 March 26^th^. See also *J Comput Chem*. 25(13):1605-12).
(TIF)
