# Supplementary figures and images for: Dimerization Interface of 3-Hydroxyacyl-CoA Dehydrogenase Tunes the Formation of Its Catalytic Intermediate
Source: PLoS One. 2014 Apr 24;9(4):e95965. doi: 10.1371/journal.pone.0095965 (PMC3999109; doi:10.1371/journal.pone.0095965)

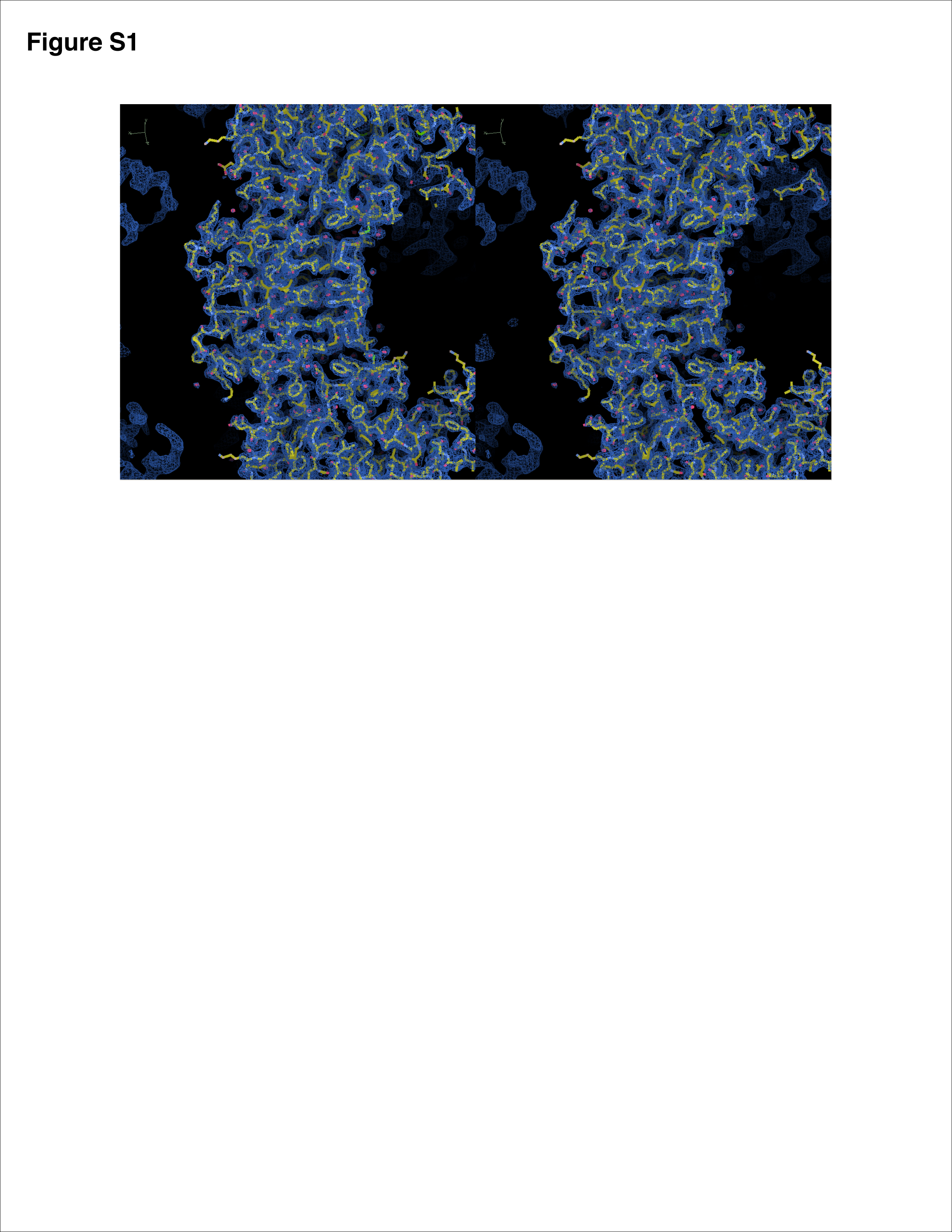

Supplement: Figure S1 — A stereo image of overall 2Fo-Fc electron density map of c HAD dimer contoured at 1.2 σ. (TIF) [file pone.0095965.s001.tif]

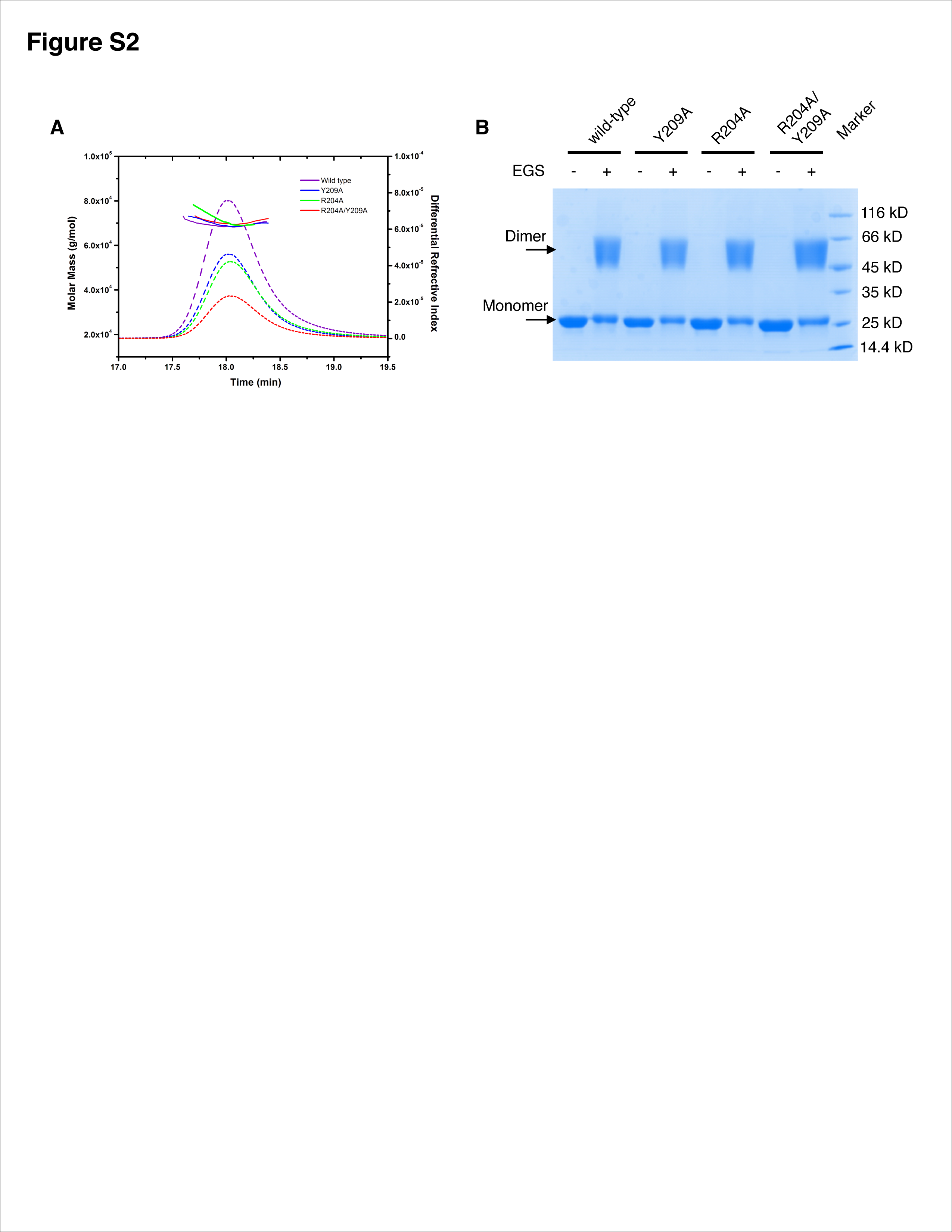

Supplement: Figure S2 — Evaluations of c HAD dimerization state in solution. (A) Molecular weight of oligomeric cHAD and its mutants. Aligned SEC-LS/UV/RI differential refractive index chromatograms (right axis) of wild type, R204A, Y209A and R204A/Y209A cHADs are represented in purple, green, blue and red dots, respectively. The molecular weights calculated according to LS and RI measurements at each time point are plotted with the scale at the left axis. (B) SDS-PAGE analysis of the EGS (Ethylene glycolbis) cross-linked cHAD and mutants. (TIF) [file pone.0095965.s002.tif]

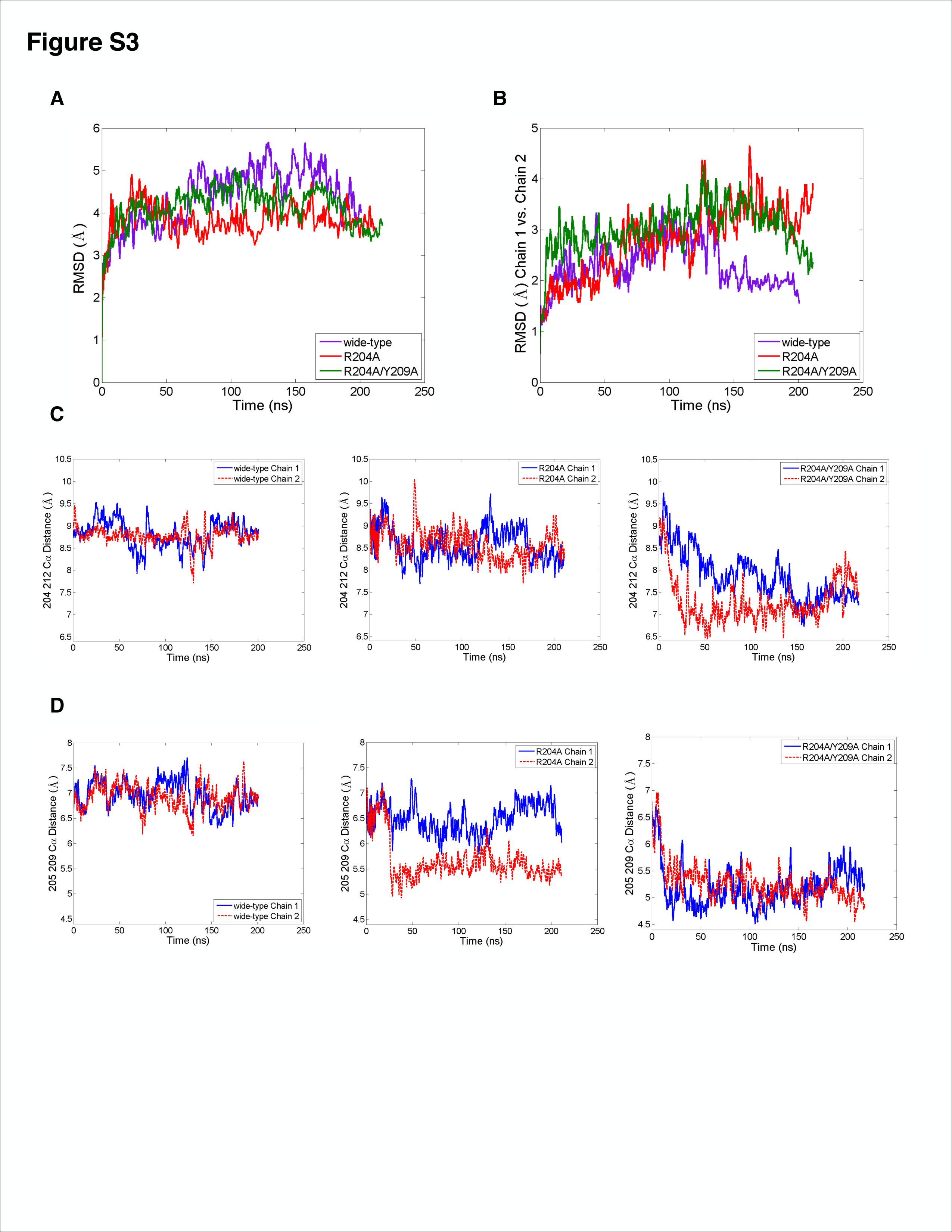

Supplement: Figure S3 — Molecular dynamics simulations of c HAD. (A) The time evolution of root-mean-square displacements (RMSD) of cHAD dimer. Curves for the wide-type, R204A and R204A/Y204A mutants are in purple, green and red, respectively. (B) The time evolution plots of RMSD of two subunits (chain 1 and chain 2) within the cHAD dimer. (C) The time evolution plots of the distances of Cα atoms of residue 204 (in chain 1/2) and residue 212 (in chain 2/1) for the wild type cHAD dimer (left) and its mutants R204A (middel) and R204A/Y209A (right). (D) The time evolution plots of the distances of Cα atoms of residue 205 (in chain 1/2) and residue 209 (in chain 2/1) for the wild type cHAD dimer (left) and its mutants R204A (middel) and R204A/Y209A (right). See also Fig. 3C and Table 4 . (TIF) [file pone.0095965.s003.tif]

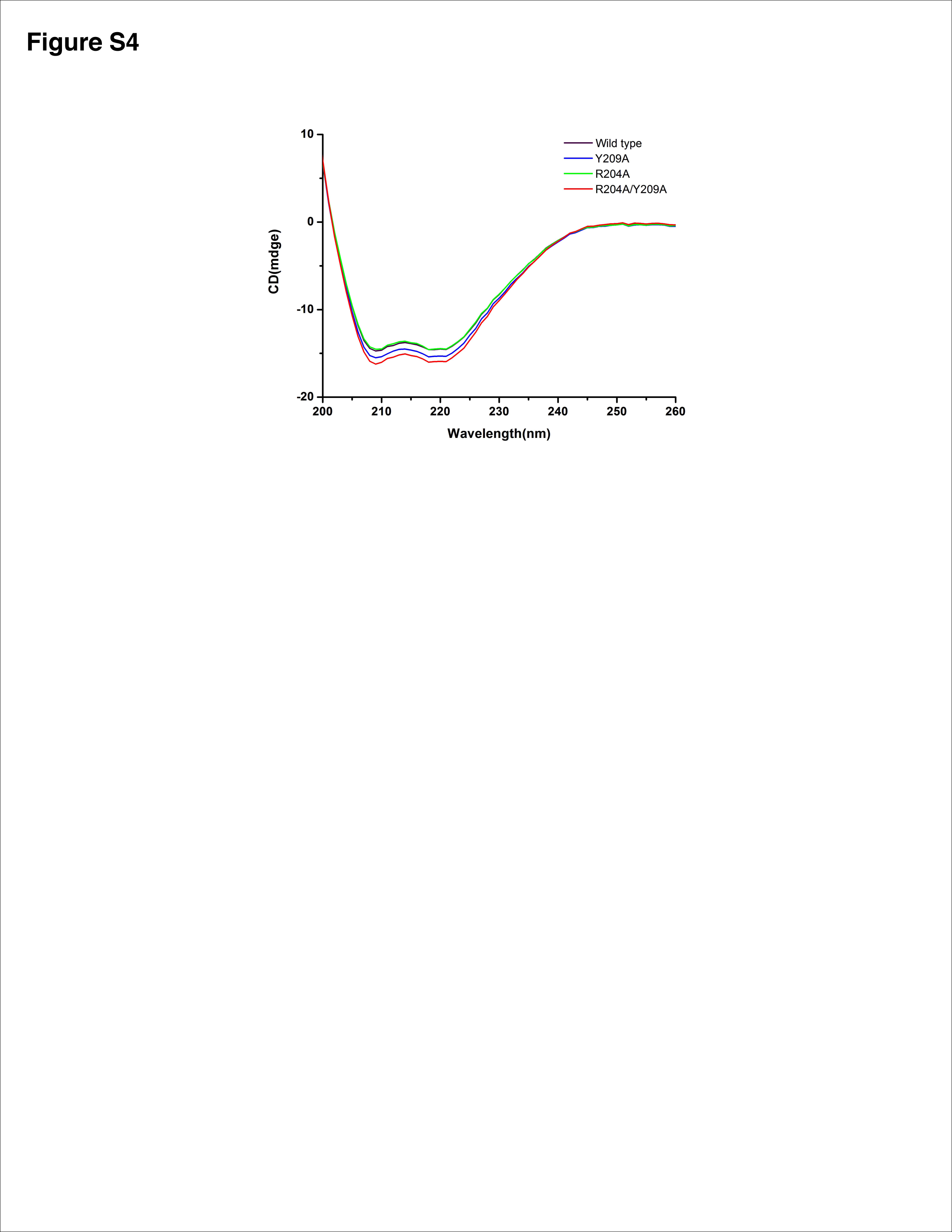

Supplement: Figure S4 — Circular dichroism (CD) spectra of the wild type and mutated c HADs. All the measurements were repeated three times and the spectrum data were corrected by subtracting the buffer control. (TIF) [file pone.0095965.s004.tif]

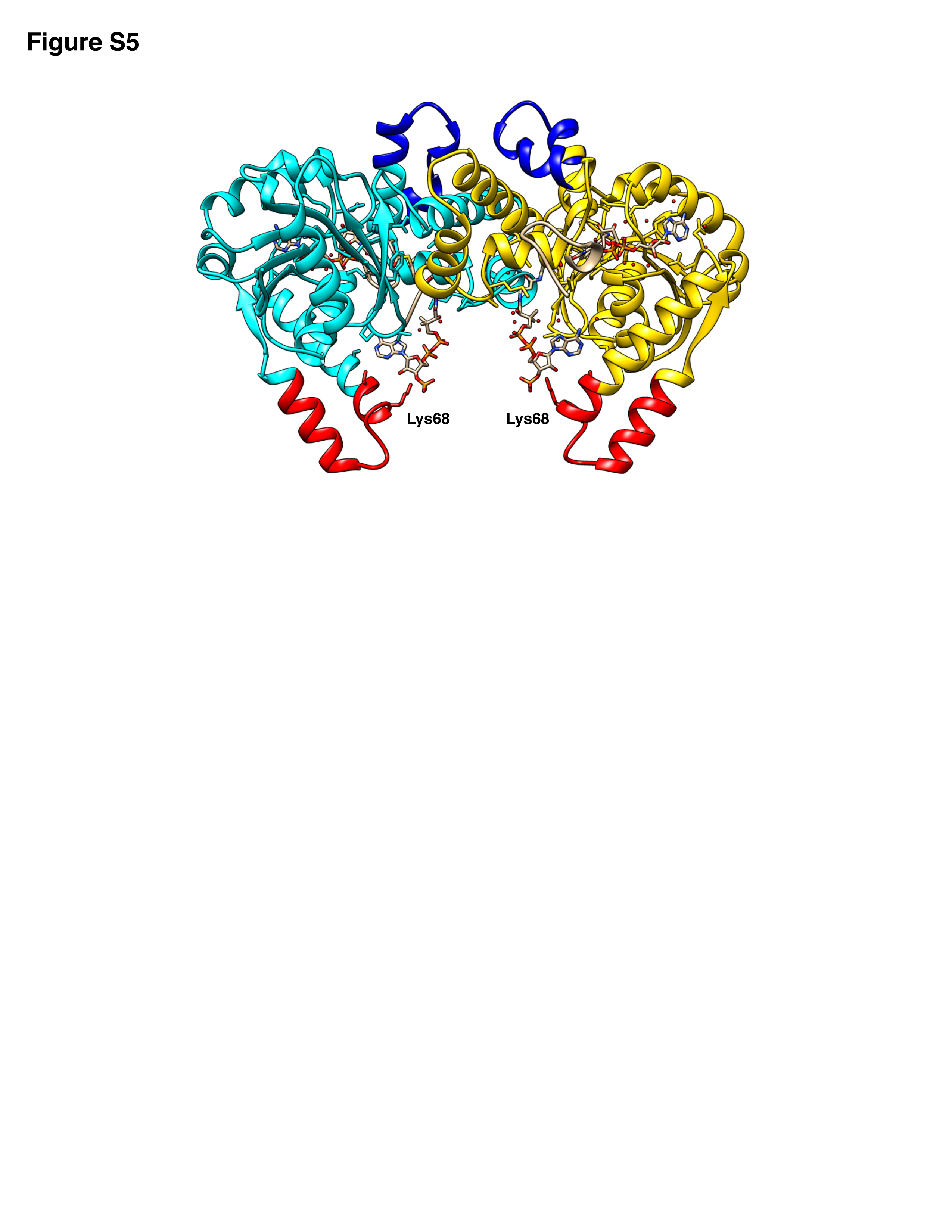

Supplement: Figure S5 — Cartoon representation of the crystal structure of human HAD ternary complex (PDB entry 1F0Y). One subunit is colored in cyan and another in gold. The regions corresponding to the regions R1 and R2 in cHAD are colored in red and blue, respectively. The cofactor NAD, substrate AACoA and the residues involved in substrate and cofactor binding are shown in stick model. This figure was prepared using UCSF Chimera (http://www.cgl.ucsf.edu/chimera/. Accessed at 2014 March 26th. See also J Comput Chem. 25(13):1605-12). (TIF) [file pone.0095965.s005.tif]
